# Assessment of willingness to Tele-monitoring interventions in patients with type 2 diabetes and/or hypertension in the public primary healthcare setting

**DOI:** 10.1186/s12911-020-1024-4

**Published:** 2020-01-28

**Authors:** David Yang Ern Sin, Xiaoxuan Guo, Dayna Wei Wei Yong, Tian Yu Qiu, Peter Kirm Seng Moey, Muller-Riemenschneider Falk, Ngiap Chuan Tan

**Affiliations:** 1 Ministry of Health Holding, Singapore, Singapore; 2 SingHealth Polyclinics, Connection One, Tower 5, #15-10, 167, Jalan Bukit Merah, Singapore, 150167 Singapore; 3 SingHealth-Duke NUS Family Medicine Academic Clinical Programme, Singapore, Singapore; 4 Yong Loo Lin School of Medicine, National University of Singapore, Singapore, Singapore

**Keywords:** Tele-monitoring, Health information technology, Model, Type-2 diabetes mellitus, Hypertension

## Abstract

**Background:**

Tele-monitoring (TM) is remote monitoring of individuals via info-communication technology, enabling them and their relatives or care-providers to recognize their health status conveniently. TM will be successful only if the individuals, often patients with medical conditions, are willing to accept and adopt it in their daily lives. This study aimed to determine the prevalence of willingness of patients with type 2 diabetes mellitus (T2DM) and/or hypertension towards the use of TM, and the factors influencing their uptake.

**Methods:**

A cross-sectional survey was conducted at two public primary care clinics (polyclinics) in north-eastern Singapore, where TM had not been implemented. After the patients with T2DM and/or hypertension consented after fulfilling the eligibility criteria, they were first introduced to the concept of TM using pictogram and explanation by the investigators. Data on their demography, clinical parameters, technological literacy and acceptance of TM based on the Health Information Technology Acceptance Model (HITAM) were subsequently collected, computed, analyzed, followed by regression analyses to identify the factors associated with their willingness to use TM.

**Results:**

Among 1125 eligible multi-ethnic Asian patients approached, 899 of them completed the assisted questionnaire survey, yielding a response rate of 79.9%. Their mean age was 58 ± 8 years, females 51.3% and Chinese 69.3%. Overall, 53.0% of the patients were willing to use TM. Personal beliefs on technology (OR = 3.54, 95%CI = 2.50–4.50, *p* < 0.001), prior technology utility (OR = 3.18, 95%CI = 1.57–6.42, *p* = 0.001), Patient’s requirements to be accompanied (OR = 1.48, 95% CI = 1.054–2.082, *P* = 0.03) Cost considerations (OR = 2.96, 95% CI = 2.257–3.388, *P* < 0.01) and technological literacy (OR = 2.77, 95%CI = 2.05–3.38, *p* < 0.001) were associated with willingness to use TM.

**Conclusion:**

Slightly over half of the patients were willing to use TM. Factors such as age, ethnicity, technological literacy, beliefs and previous utility of technology of the patients have to be addressed before implementing TM in primary care.

## Background

Telemedicine or Telehealth is the delivery of healthcare services remotely by means of telecommunications technology [1, 2]. Tele-monitoring (TM is a method of remote monitoring of vital parameters by persons outside of healthcare setting (such as from their residences), which are transmitted electronically via blue tooth technology to the healthcare provider [3–8]. Leveraging on TM to check on the clinical status of patients with non-communicable diseases (NCD) such as Type 2 Diabetes mellitus (T2DM) and hypertension in the community is of particular significance and relevance to their optimal long term management. The global prevalence of NCD is increasing rapidly. T2DM was estimated to be 9% in 2014, contributing to 1.5 million deaths and 89 million disability-adjusted life-years (DALYS) [9]. Global prevalence for hypertension was even higher at over 22%, leading to 9.4 million deaths [1, 9].

There is thus an urgent need to curb the rising morbidity and mortality from these NCD. Traditional model of care for NCD is episodic. Physicians review patients during face-to-face consultations but do not have access to their clinical status in between consultations. Patients may develop complications as a result of events or occurrences that go unnoticed or unattended to during this interval, resulting in missed opportunities for early intervention to prevent adverse outcomes.

Optimal glycaemic and blood pressure (BP) control are critical in preventing vascular complications and mortality. Regular monitoring of these vital parameters via TM allows patients to gain better insights into their real-time diabetes and hypertension control in relation to their treatment. TM has shown to be beneficial for chronic disease management [3–8] such as in Diabetes mellitus management in children, outcomes in women with gestational Diabetes Mellitus, in management of uncontrolled hypertension and paired with self-monitoring of blood pressure. In addition, any abnormal health status trend detected via TM can alert the patients and their care providers, enabling them to take remedial measures to prevent complications. Thus, TM extends the scope of patient monitoring beyond the clinical setting, which is important for surveillance of NCD such as diabetes mellitus and hypertension.

Acceptance and adoption of TM is pivotal in achieving its objectives. Despite its potential to complement traditional model of care, the benefits of TM can only be reaped if there is sufficient uptake by patients themselves. Theoretical frameworks have been developed to assess the acceptance of technology implementation in healthcare, one of which is the Health Information Technology Acceptance Model (HITAM) [10]. This framework takes into account behavioural beliefs, normative beliefs and efficacy belief, leading to the concepts of perceived threat, usefulness and ease of use respectively.

Understanding and addressing the barriers towards the use of TM is necessary for its successful implementation. Known deterrents of TM include older age, lower education, patients’ beliefs, concerns about the costs and privacy, and preferences for face-to-face consultations [11–16].

These factors are influenced by the socioeconomic, psychological and cultural context of the local population. Located at the centre of Southeast Asia, Singapore has an urbanised, Western-educated multi-ethnic Asian population with one of the highest penetrance of info-communication technology in the world [17, 18]. Nonetheless, a local study in 2008 reported that only 40.3% of patients, who were managed in public primary care centres (polyclinics), were willing to use communication technologies in healthcare services, namely Short Message Service (SMS) and internet [19]. Patients’ personal beliefs and technological literacy were the main factors which influenced their acceptance of a technology-based healthcare system. However, we postulated that the level of acceptance of TM would have risen since 2008, as info-communication technology has advanced significantly over the last decade to mitigate some of the barriers.

The need to re-evaluate the acceptability and perceptions of TM in Singapore is imminent, in view of rising prevalence of NCD such as T2DM and hypertension, and a shrinking workforce to support its rapidly aging population [20]. In the last 10 years, complexity of technology, access to technology, health behaviors and the medico-legal landscape have significantly evolved [21]. .Population demographics and healthcare needs in Singapore have also evolved in the past decade, with an aging population increasingly consisting of more educated and self-reliant middle-aged population, and a shift in focus from tertiary and acute care to preventive and primary care in the community. Since 2008, there have been greater integration of technology and healthcare such as through E-appointments, E-payment, Teleradiology at the institution where the study was executed. However, technology can be expensive. With rising number of people living with chronic diseases, increasing healthcare cost and increasing evidence to support the use of tele-monitoring in patients with non-communicable diseases, a re-evaluation of the acceptability and perceptions of Tele-monitoring in Singapore is timely to support its implementation successfully in the community, so as to maximize the use of scarce healthcare manpower and resources.. TM is a potential tool to maximise the healthcare manpower to manage these NCD and to engage the respective patients in self-management to optimise disease control and alleviate the rising disease burden. Thus the study aimed to determine the prevalence of willingness and associated factors in the use of TM by patients who consulted the polyclinics for their T2DM and/or hypertension management. It takes into consideration that patients have varying levels of understanding of tele-monitoring, and that tele-monitoring interventions, with the potential to be wide-ranging, are grounded by similar concepts.

## Method

### Study design

**Fig. 1:**
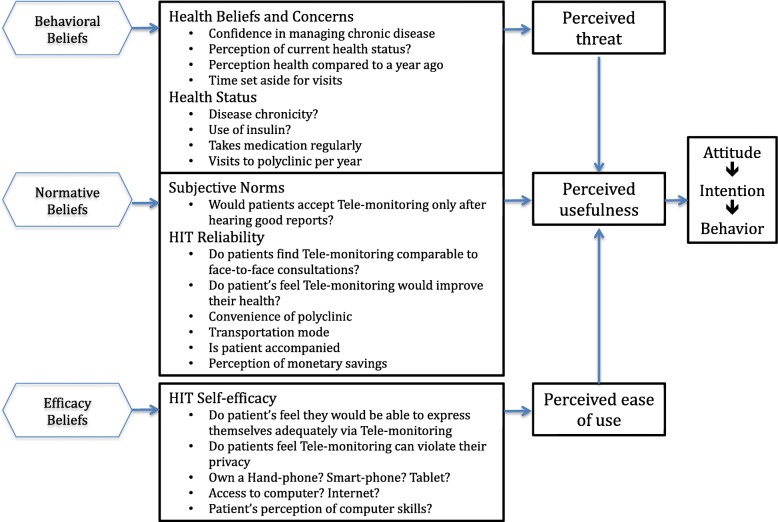
HITAM Model with the relevant questions used in this study, adapted from: Kim J et al. [4]

A cross-sectional observational study was conducted using interviewer-administered questionnaires. The questionnaires consists of 3 segments. First, information was collected on patients’ biodata, perceptions towards medical conditions, self-monitoring behaviours, accessibility to technology and technological literacy, underpinned by the Health Information Technology Acceptance Model (HITAM) [10, 22–33]. (Additional file 1: Questionnaire and Fig. 1).

**Fig. 2:**
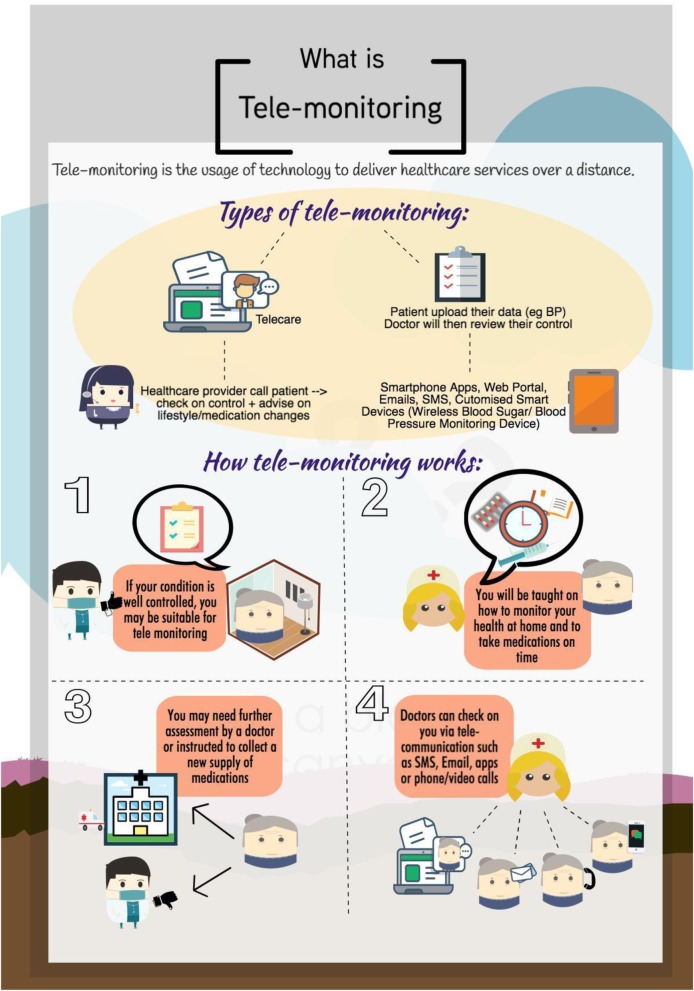
Infographic on Tele-monitoring to educate participants

Next, concepts and current TM services were introduced to each patient via a scripted explanation and information pamphlet to ensure a standardised understanding of TM across the study. (Figure 2 in Appendix): education segment. The information were standardized and scripted after close discussion with SingHealth Polyclinics, a major public primary healthcare provider.

In the final segment, the willingness to use TM and associated factors were assessed based on the question “Would you be willing to use tele-monitoring as part of managing your diabetes/ hypertension now?” Response options to this question were “Yes”, “Sometimes”, “No”, and “Unsure”. As TM will require a certain level of commitment to work as intended, a “yes” response was taken to mean that the patient was willing, while the remaining options were regarded as unwilling.

A pilot study was conducted at a clinic to assess the content of the questionnaire as well as the feasibility of its administration. Amendments were made based on the feedbacks and issues identified during the pilot study. The final questionnaire was also translated to mandarin language for ease of administration to the predominant Chinese patient pool in the polyclinics.

### Study site and population

Multi-ethnic Asian patients were recruited from two public primary care clinics (polyclinics) located in Pasir Ris and Sengkang in north-eastern Singapore [34]. In 2015, the former manages daily attendances of 600–700 patients in an estate with a population of 139,890 residents. The latter attended to 1000–1100 patients daily in an estate populated by 206,690 residents.

The eligibility criteria for study participants were: aged 21–70 years old; had Type 2 DM and/or hypertension with ≥2 follow-up consultations within the past year and were able to converse in English or Mandarin. We excluded patients with prior exposure to TM and/or could not respond to the survey due to language, cognitive, hearing or visual barriers.

### Recruitment

This study used consecutive sampling to recruit suitable patients. Study team members were stationed at the Health Monitoring Station (HMS) in each study site, where all patients on follow-up visits for diabetes and/or hypertension had measurements of their blood pressure and body mass index by trained healthcare workers. Every patient at the HMS from 7 to 15 February 2017 were approached and screened for their eligibility for this study.

After queries on the study were addressed, verbal consent was obtained from each patient in a private room where the questionnaire survey was conducted. A participant information sheet was provided for all participants. A total of 37 interviewers, comprising final year medical students, underwent rigorous pre-implementation training prior to the study initiation. Every interviewer used the standardized script to administer the questionnaire to ensure consistency.

### Sample size estimation and statistical analysis

Assuming a 3% error margin and a prevalence of willingness of 66.8% derived from studies done overseas, it was estimated that a total of 944 respondents would be required [35–37]. All statistical analyses were performed using IBM SPSS Statistics software Version 23.0 (IBM Co., Armonk, New York, US). Descriptive data is presented as counts and in percentages. Bivariate analyses were conducted using the Pearson Chi-Squared Test for categorical variables. To adjust for confounding variables, a logistic regression model was performed on all factors that were significant in the univariate analysis. A *p*-value of < 0.05 was used to determine statistical significance.

### Ethics approval and funding

This study was approved by SingHealth Centralised Institutional Review Board (CIRB Ref: 2016/3162). Funding for printing of questionnaires and materials was provided by the Yong Loo Lin School of Medicine at the National University of Singapore. The investigators declared no conflict of interest in this study.

## Results

Among the 1125 eligible patients who were approached, 899 of them consented and completed the survey, yielding a response rate of 79.9%. The demographic characteristics of the study population are presented in Table 1.

**Table 1:** Socio-demographic characteristics of patients and their willingness to use TM

	Total, n (%)	Willing^a^, n (%)	Odds ratio and confidence Interval	*p* value
All participants	899 (100)	472 (52.5)	NA	NA
Gender				
Female	460 (51.2)	224 (48.7)	Reference	0.02
Male	439 (48.8)	248 (56.5)	1.367 (1.051–1.779)	
Age				
≤ 59	465 (51.7)	278 (59.8)	1.838 (1.410–2.398)	< 0.01
60 and older	434 (48.3)	194 (44.7)	Reference	
Age (10 year intervals)				
≤ 40	22 (2.4)	17 (77.3)		< 0.01
41–50	115 (12.8)	78 (67.8)		
51–60	382 (42.5)	209 (54.7)		
61–70	380 (42.3)	168 (44.2)		
Ethnicity				
Chinese	626 (69.6)	305 (48.7)	Reference	
Non-Chinese	273 (30.4)	167 (61.2)	1.658 (1.240–2.212)	< 0.01
Spoken language				
Chinese	329 (36.6)	130 (39.5)	Reference	
English	570 (63.4)	342 (60.0)	2.293 (1.739–3.030)	< 0.01
Marital status^a^	895 (100)	471 (52.6)		
Single	171 (19.1)	77 (45.0)	Reference	
Married	724 (80.9)	394 (54.4)	1.458 (1.043–2.037)	0.03
Employment status^a^	892 (100)	469 (52.6)		
Working	538 (60.3)	306 (56.9)	1.546 (1.180–2.024)	< 0.01
Not working	354 (39.7)	163 (46.0)	Reference	
Highest education level				
None/ PSLE	231 (25.7)	92 (39.8)	Reference	
O-Level and higher	668 (74.3)	380 (56.9)	1.994 (1.470–2.704)	< 0.01
Total household income^a^ (S$)	668 (100)	378 (56.6)		
< 3400	317 (47.5)	162 (51.1)	Reference	
≥ 3400	351 (52.5)	216 (61.5)	1.531 (1.125–2.083)	< 0.01
Financial assistance^a^	895 (100)	470 (52.5)		
Yes	392 (43.8)	182 (46.4)	Reference	
No	503 (56.2)	288 (57.3)	1.545 (1.184–2.016)	< 0.01

^a^n for these factors are < 899 instead of the total n = 889 participants as some individuals deemed these questions sensitive and declined to answer.

### Primary study objective: willingness to take-up Tele-monitoring

A total of 472 patients (52.5%) were willing to take up TM to manage their condition, regardless of their medical conditions. Among the 427 participants who reported unwillingness to use TM, 52.2% of them felt that they would be willing after hearing positive reports.

### Factors associated with willingness to take up Tele-monitoring

All socio-demographic characteristics investigated were significantly associated with willingness to take up TM based on univariate analysis. Younger, males, married, minority ethnicity, English speaking, working patients, those with higher education, those with higher income and those without any need for financial assistance were more willing to use TM (Table 1).

Table 2 shows the HITAM related factors which were associated with willingness to use TM. Hand-phone and smartphone ownerships, and self-reported computer skills were associated with increased willingness to use TM. In contrast, patients who set aside more time for polyclinic visits, and those who had concerns about privacy violations were less willing to use TM.

Results of the multi-variate analysis are presented in Table 3. Patients who perceived comparable satisfaction between TM and face-to face physician consultation, no privacy violation, cost savings with TM, need accompaniment for physician visit, were convinced of positive results of TM, and did not face challenges in using communication devices, were associated with willingness to take up TM.

**Table 3:** Factors associated with willingness to use TM

Factors associated with willingness	*p*-value	Odds ratio
Gender	0.44	0.875 (0.622–1.231)
Age	0.29	0.826 (0.579–1.178)
Ethnicity	0.13	0.743 (0.507–1.087)
Highest Education	0.30	1.271 (0.806–2.004)
Employment	0.41	1.164 (0.811–1.671)
Marital Status	0.94	1.018 (0.669–1.548)
Financial Assistance	0.18	0.794 (0.567–1.112)
(Perception of Health) In general, would you say your health is:	0.21	1.240 (0.886–1.736)
How much time did the patient set aside for the appointment today	0.32	0.821 (0.556–1.214)
Perception of health compared to 1 year ago^a^	0.70	1.082 (0.723–1.620)
Tele-monitoring would be satisfactory compared to seeing the doctor in person.	< 0.01	2.790 (1.961–3.970)
Is patient accompanied	0.04	1.595 (1.029–2.473)
Perceptions on monetary savings from telemedicine^a^	0.01	1.777 (1.279–2.469)
Patient would be more convinced after seeing benefits from reports	0.04	1.425 (1.019–1.994)
Tele-monitoring can violate patients’ privacy	0.02	0.635 (0.432–0.934)
Handphone^a^	0.67	0.483 (0.017–13.359)
Access to computer^a^	0.22	1.323 (0.841–2.082)
Access to internet	0.41	0.748 (0.375–1.493)
Computer skills^a^	0.11	1.480 (0.913–2.401)
Use Smartphone apps^a^	0.34	1.235 (0.798–1.911)
Tablet	0.08	1.352 (0.968–1.890)
Patient feels communication devices too challenging	0.02	1.546 (1.088–2.192)
Patient is concerned they are unable to express their problems over telemonitoring	0.06	1.585 (1.139–2.203)

^a^n for these factors are < 899 instead of the total n = 889 participants as the question was not applicable to the participant or they declined to answer.

## Discussion

Our study revealed a slim majority of our study population (52.5%) were willing to use TM to manage their NCD. Nonetheless, the willingness to use TM has increased compared to 40.3% in the earlier survey [19]. In this study 95.1 and 88.5% of patients owned a hand-phone and had access to the internet respectively, versus 71.2 and 49.4% in the earlier study.

Greater access to mobile technology is postulated to be associated with the rising willingness to use TM. Based on HITAM, ownership of hand-phone(s), usage of smartphone apps and having at least basic computer skills were important ‘technological’ factors influencing the use of TM (Table 2). Those who found “using communication devices challenging” were less willing to use TM (Table 3). Nevertheless, over these years, government agencies have launched programmes, such as the Smart Nation Singapore initiative, to improve the technology literacy of the local population [38]. Specific efforts are directed to equip the elderly with the relevant technological skills through various free or heavily subsidized courses and workshops in the community [39]. The prevalence of willingness to use TM in disease surveillance is expected to escalate in the immediate future with these measures to lower the barriers [19].

**Table 2:** HITAM-related factors influencing willingness to use TM (Univariate analysis)

	Total, *n*(%)	Willing^a^, *n*(%)	Odds ratio and confidence Interval	*P* Value
Behavioural Beliefs (Health Status)				
No. of Years since diagnosis of diabetes mellitus^a^				
5 or less	210 (49.5)	123 (58.6)	1.672 (1.139–2.457)	< 0.01
More than 5 years	214 (50.5%	98 (45.8)	Reference	
No. of Years since diagnosis of hypertension^a^				
5 or less	298 (40.7)	167 (56.0)	1.298 (1.020–1.745)	0.10
More than 5 years	434 (59.3)	215 (49.5)	Reference	
Is patient on insulin injection^a^				
On insulin	49 (11.3)	27 (55.1)	1.165 (0.641–2.117)	0.65
Not on insulin	384 (88.7)	197 (51.3)	Reference	
T2DM and Hypertension medication^a^				
Takes regularly	849 (94.6)	449 (52.9)	1.220 (0.682–2.184)	0.55
Does not take regularly	48 (5.4)	23 (47.9)	Reference	
Visits to Polyclinic per year^a^				
4 or less visits	587 (65.5)	296 (62.7)	1.301 (0.986–1.717)	0.07
More than 4 visits	309 (34.5)	176 (57.0)	Reference	
Behavioural beliefs (health beliefs and concerns)				
In general, would you say your health is (i.e. perception of health):				
Good	504 (56.1)	293 (58.1)	1.676 (1.285–2.185)	< 0.01
Poor	395 (43.9)	179 (45.3)	Reference	
How much time did the patient set aside for the appointment today^a^				
< 3 h	705 (78.5)	385 (54.6)	1.427 (1.036–1.964)	0.03
> 3 Hours	193 (21.5)	88 (45.6)	Reference	
Confidence managing T2DM/Hypertension				
Confident	630 (70.1)	350 (74.2)	1.506 (1.130–2.007)	0.06
Not confident	269 (29.9)	122 (45.4)	Reference	
Perception of health compared to 1 year ago^a^				
Worse health	197 (21.9)	87 (44.2)	Reference	
Same or better	701 (78.1)	384 (54.7)	1.532 (1.114–2.105)	0.01
Normative beliefs (hit reliability)				
TM would be satisfactory compared to seeing the doctor in person^a^				
Agree	316 (35.1)	225 (71.2)	3.535 (2.5–4.449)	< 0.01
Disagree	582 (64.9)	247 (42.4)	Reference	
Convenience of visit to polyclinic				
Convenient	825 (91.8)	435 (52.7)	1.115 (0.693–1.795)	0.72
Not convenient	74 (8.2)	37 (50.0)	Reference	
Transport Mode†				
Public or personal transport	626 (72.2)	334 (53.4)	1.116 (0.829–1.502)	0.50
Walk	241 (27.8)	122 (50.6)	Reference	
Is patient accompanied				
Accompanied	169 (18.8)	102 (60.4)	1.481 (1.054–2.082)	0.03
Not accompanied	730 (81.2)	370 (50.7)	Reference	
Perceptions on monetary savings from telemedicine^a^				
Saves money	470 (52.4)	306 (65.1)	2.963 (2.257–3.388)	< 0.01
Does not save money	427 (47.6)	165 (38.6)	Reference	
Normative beliefs (Subjective Norms)				
Patient would be more convinced after seeing benefits from reports				
More willing	532 (59.2)	309 (58.1)	1.734 (1.326–2.268)	< 0.01
Not more willing	367 (40.8)	163 (44.4)	Reference	
Efficacy beliefs (hit self efficacy)				
Patients feel they would not be able to explain their problems adequately via tele-monitoring.^a^				
Disagree	465 (51.8)	203 (43.7)	Reference	
Agree	433 (48.2)	268 (61.9)	2.096 (1.605–2.740)	< 0.01
Tele-monitoring can violate patients’ privacy				
Agree	204 (22.7)	90 (44.1)	Reference	
Disagree	695 (77.3)	382 (55.0)	1.546 (1.129–2.119)	< 0.01
Handphone^a^				
Owns	857 (95.4)	461 (53.8)	3.175 (1.571–6.418)	< 0.01
Does not own	41 (4.6)	11 (26.8)	Reference	
Smartphone^a^				
Owns	778 (86.6)	433 (55.7)	2.607 (1.735–3.917)	< 0.01
Does not own	120 (13.4)	39 (32.5)	Reference	
Access to computer^a^				
Has access to computer	666 (74.2)	384 (57.7)	2.228 (1.640–3.027)	< 0.01
No access to computer	232 (25.8)	88 (37.9)	Reference	
Access to internet				
Yes	792 (88.4)	435 (54.9)	2.206 (1.442–3.375)	< 0.01
No	232 (11.6)	37 (15.9)	Reference	
Computer skills^a^				
Yes	635 (70.6)	380 (59.8)	2.77 (2.054–3.375)	< 0.01
No	262 (29.4)	93 (35.5)	Reference	
Use Smartphone apps^a^				
Uses apps	609 (78)	366 (60.1)	2.36 (1.669–3.339)	< 0.01
Does not use apps	172 (22)	67 (39.0)	Reference	
Tablets				
Owns	407 (45.5)	252 (61.9)	2.023 (1.547–2.645)	< 0.01
Does not own	487 (54.5)	217 (44.6)	Reference	
Patient feels communication devices too challenging				
Challenging	397 (44.2)	160 (40.3)	Reference	
Not Challenging	502 (55.8)	312 (62.2)	2.227 (1.859–3.185)	< 0.01

^a^*n* for these factors are < 899 instead of the total *n* = 889 participants as the question was not applicable to the participant or they declined to answer

Patients who required caregivers to accompany them for their physician consultation were more willing to use TM. Convenience is likely the major attributing factor. For stable patients who are diligently monitoring their clinical parameters, TM could potentially replace a physician visit but safety has to be evaluated more stringently with appropriately designed trial. The healthcare infrastructure must also be able to document their health status via linkage to the electronic health records and support the continuity and timely supply of their medications. However, TM should not be confused with tele-consultation. It should remain as an adjunct tool to support patients in their chronic disease management.

Individual beliefs and perceptions of TM in healthcare also influence a patient’s willingness to use TM. Patients were more willing to use TM if they were shown reported evidences of its effectiveness. However, the results showed that patients had other concerns.

The fee-for-service healthcare system in Singapore also has implications for the introduction of TM to patients. Patients who consult primary care physicians have to pay for the services and medications. The technology involved in TM can be expensive and cost will eventually be passed over to these primary care users. What mattered to them seemed to be their perceived cost-savings of using the TM system, as alluded in this study. Cost consideration for adding on TM to a polyclinic consultation is inevitable. It is imperative to assess if the population is ready to pay for the new technology in monitoring their health. Substitution for a face-to-face consultation when clinical parameters from the TM system are stable and economy of scale when the pool of users expands can potentially lead to cost-savings. Thus, aside from patients, the results of the study will interest the health finance and policy makers to evaluate the feasibility and cost-effectiveness of scaling up a TM system to support chronic disease management nation-wide.

These factors are potentially modifiable but will require time and intervention to gain public trust. TM has been implemented in isolated private healthcare system in Singapore but the results are not published [40]. A well-designed randomised controlled trial on TM is being planned in a public primary care institution, which will be able to provide proof of values in terms of satisfaction of TM to the users, its cost-effectiveness and scalability in its implementation.

Perception of non-intrusion to personal privacy in using TM is also a key influencing factor. A TM system with secured data encryption, data repository and restricted access when transmitting patient data to healthcare providers [41] is essential for its implementation [42]. .Privacy protection is especially critical where multiple incidences of data breaches in local healthcare systems were recently reported [43, 44]. Local healthcare providers have to work closely with the official IT governing agencies to ensure strict adherence to measures which have since been implemented for personal data protection.

The strength of this study lies in the systematic sampling of the potential patients, notwithstanding the large daily volumes of patients at the study sites. The results showed that perceptions and beliefs of TM influenced the willingness of its adoption. They are modifiable and can be mitigated with appropriate measures to optimise the successful implementation of TM in primary care.

Nonetheless, we acknowledged that self-reported willingness to adopt TM might not correspond to actual utility by the patients. The forthcoming trial will provide more objective evidences of TM utilization.

## Conclusion

Our study showed that 52.5% of patients with T2DM and/or hypertension reported willingness to adopt TM. They were influenced by perceived effectiveness ease of use, cost savings, privacy protection, and satisfaction of care using TM compared to face-to-face physician consultation. It is heartening to recognise from the results that the awareness of telemonitoring among patients has increased with the growth and expanding use of technology in our society. A randomised controlled study (RCT) is to be implemented soon, to prove effectiveness in health outcomes of the patients recruited into the telemonitoring system compared with usual care. Beyond the RCT, there are plans to scale up the telemonitoring system to cater to those who are currently unwilling to use it using implementation science approaches.

### Supplementary information

**Additional file 1.** Questionnaire

## Data Availability

The datasets used and analysed during the current study are available from the corresponding author on reasonable request.

## Abbreviations

BP: Blood pressure
DALYS: Disability-adjusted life-years
HITAM: Health Information Technology Acceptance Model
HMS: Health Monitoring Station
NCD: Non-communicable diseases
SMS: Short Message Service
T2DM: Type 2 Diabetes mellitus
TM: Tele-monitoring
