# Supplementary material for: Assessment of willingness to Tele-monitoring interventions in patients with type 2 diabetes and/or hypertension in the public primary healthcare setting
Source: BMC Med Inform Decis Mak. 2020 Jan 28;20:11. doi: 10.1186/s12911-020-1024-4 (PMC6986094; doi:10.1186/s12911-020-1024-4)
Supplement: Supplementary file 1 — Additional file 1. Questionnaire [file 12911_2020_1024_MOESM1_ESM.docx]

**Additional file 1: Questionnaire**

**For Student Administrators Only:**

| **Serial Number:** | **Assessment of willingness to use tele-monitoring interventions in patients with diabetes mellitus and hypertension in the public primary healthcare setting** | | | | **Language:**  English |
| --- | --- | --- | --- | --- | --- |
| **Student Administrator:** | | **Date of survey:**  **Time of survey:** | **Polyclinic:** | **Polyclinic Location:** | |

**Participant Survey:**

1. Gender:

⎕ Male ⎕ Female

1. Age (years):

______

1. Ethnic group:

⎕ Chinese ⎕ Malay ⎕ Indian ⎕ Others

1. Highest Educational Qualification Attained:

⎕No formal education/PSLE ⎕ Secondary, GCE ‘O’ / ‘N’ level

⎕ GCE ‘A’ level / Diploma ⎕ Degree / Professional qualification

1. Current employment status:

⎕ Home duties ⎕ Unemployed ⎕ Manual labour ⎕ Sales/Office work

⎕ Professional ⎕ Retired ⎕ Pensioner ⎕ Self-employed

1. Monthly Household Income (Exclusive of CPF):

⎕ Less than S$1,150 ⎕ S$1,150 – S$2,250 ⎕ S$2,251 – S$3,400 ⎕ S$3,401 – S$5,650 ⎕ S$5,651 – S$8,750 ⎕ S$8,751 – S$11,250 ⎕ S$11,251 – S$16,650

⎕ S$16,651 – S$22,500 ⎕ More than S$22,500 ⎕ I choose not to declare ⎕ I don’t know

1. How many people are there in your household, including yourself? (by household we mean those persons who are normally resident or share expenses)

No. of persons in household ______

1. Financial Assistance Scheme:

⎕ CHAS (Blue) ⎕ CHAS (Orange) ⎕ Pioneer Generation ⎕ Others：___________

⎕ None

1. Marital Status:

⎕ Single ⎕ Married ⎕ Widowed ⎕ Divorced/Separated

⎕ I choose not to declare

**Technology literacy**

1. Do you have a handphone?

⎕ Yes ⎕ No

1. Do you have a smartphone?

⎕ Yes ⎕ No (Proceed to Q15) ⎕ I don’t know (Proceed to Q15)

1. What is the operating system of your smartphone?

⎕ iOS (Apple) ⎕ Android ⎕ Windows ⎕ Others:__________

1. Do you use smartphone apps?
   ⎕ Yes ⎕ No ⎕ I do not own a smartphone
2. Do you download smartphone apps?
   ⎕ Yes ⎕ No ⎕ Someone downloads them for me
3. Do you have a tablet?

⎕ Yes ⎕ No ⎕ I don’t know

1. Do you have access to a computer?

⎕ Yes ⎕ No

1. Do you have access to Internet?

⎕ Yes ⎕ No

1. How would you rate your computer skills: (Please 🗸 **one box** only)

⎕ *Basic User* (able to do basic word processing, such as typing, and use the Internet)

⎕ *Intermediate user* (have mastered the basics and have developed additional skills, including the use of different software programs)

⎕ *Advanced user* (knowledgeable of hardware and software; able to problem-solve and advice and teach others).

⎕ I have no computer skills at this time.

**Medical conditions and attitudes/perceptions towards control**

1. Please indicate below which chronic condition(s) you have:

⎕ Diabetes Mellitus (Type 2) No. of Years since diagnosis: ____________

⎕ High blood pressure No. of Years since diagnosis: ____________

Blood Pressure today: ____________

*Please answer question 19 where applicable:*

**19A) For Diabetes Mellitus (Type 2):**

1. Do you have a blood sugar meter to monitor your blood sugar level?

Blood Sugar Meter : ⎕ Yes ⎕ No

1. How many times in a TYPICAL WEEK do you measure your blood sugar level?

Blood sugar: _____ times/week

1. What are some reasons that discourage you from self-monitoring your blood sugar? (Tick all that applies)

⎕ Test strips and needles are costly

⎕ I get frustrated by high blood sugar readings

⎕ I have a fear of needles and pain

⎕ It is inconvenient

⎕ Not interested

⎕ I lack knowledge in self-monitoring

⎕ I lack confidence in self-monitoring

⎕ I have no time

⎕ I often forget

⎕ I have received poor doctor’s instructions on how to self-monitor

⎕ My workplace is not conducive

⎕ I fear stigma from the public

⎕ Others: _________________

1. What are some motivations for self-monitoring blood sugar? (Tick all that applies)

⎕ Experiencing symptoms of low blood sugar

⎕ I feel more in control of my health

⎕ I want to please the doctor

⎕ Good family support

⎕ I want to see the effects of lifestyle changes

⎕ Others: _________________

**19B) For Hypertension:**

1. Do you have a blood pressure set to monitor your blood pressure?

Blood pressure set : ⎕ Yes ⎕ No

1. How many times in a TYPICAL WEEK do you measure your blood pressure?

Blood pressure: _____ times/week

1. What are some reasons that discourage you from home blood pressure monitoring? (Tick all that applies)

⎕ The blood pressure set is costly

⎕ I get frustrated by high blood pressure readings

⎕ It is inconvenient

⎕ Not interested

⎕ I lack knowledge in self-monitoring

⎕ I lack confidence in self-monitoring

⎕ I have no time

⎕ I often forget

⎕ I have received poor doctor’s instructions on how to self-monitor

⎕ Others: _________________

1. What are some motivations for home blood pressure monitoring? (Tick all that applies)

⎕ It is convenient

⎕ I feel empowered by self-monitoring

⎕ I want to please the doctor

⎕ Good family support

⎕ I want to see the effects of lifestyle changes

⎕ Others: _________________

1. In a typical week, did you take your medications for *Diabetes/Hypertension* regularly?

⎕ Yes ⎕ No ⎕ Don’t know

⎕ I am not on medications for Diabetes/Hypertension/Both (Circle accordingly)

1. Please specify the name of the medication you took:
2. Are you on insulin injections?

⎕ Yes ⎕ No ⎕ Not Applicable (Non-Diabetic Patient)

1. In the past 12 months, how many times did you visit a doctor? (inclusive of visits to polyclinic, private general practitioners, A&E and hospital admissions)

______ visits/year

1. How confident do you feel that you can control your *Diabetes/Hypertension* so that it does not interfere with everyday activities?

⎕Not at all confident ⎕ Not really confident ⎕ Neutral⎕ Confident ⎕ Very confident

1. In general, would you say your health is:

⎕Excellent ⎕ Very good ⎕ Good ⎕ Fair ⎕ Poor

1. **Compared to one year ago**, how would you rate your health in general **now**?

⎕Much better than one year ago ⎕ Somewhat better than one year ago

⎕ About the same ⎕ Somewhat worse than one year ago

⎕Much worse than one year ago

**Accessibility to healthcare**

1. It is convenient to travel to this polyclinic from my last location.

⎕Strongly disagree ⎕Disagree ⎕Neutral ⎕ Agree ⎕Strongly Agree

1. Mode of transport to the polyclinc
   ⎕ Walking ⎕ Bus ⎕ LRT/MRT ⎕ Taxi

⎕ Motorized Personal Transport (Car, Motorbike, Electronic Scooter, etc)

⎕ Non-Motorized Personal Transport (Cycling, etc)

1. How much time did you set aside for the appointment today?

⎕ 1h or less ⎕ 1h–3h ⎕ 3h–4h ⎕ Over 4h

1. Did someone accompany you for your appointment today?

⎕ No ⎕ Yes – it was not necessary, but for company

⎕ Yes – to help with the transport

**[Education Segment]**

**Post-education receptiveness**

1. Have you previously used tele-monitoring as a patient?

⎕ Yes ⎕ No ⎕ Not Sure

1. A) Would you be willing to use tele-monitoring as part of managing your diabetes now?
   (Please skip this question if you do not have diabetes)

⎕ Yes ⎕ Sometimes ⎕ No ⎕ Unsure

1. Would you be willing to use tele-monitoring as part of managing your hypertension now? (Please skip this question if you do not have hypertension)

⎕ Yes ⎕ Sometimes ⎕ No ⎕ Unsure

*If your answer was not “yes” for either 30A) or 30B), please answer the following questions:*

1. Would you be willing to use tele-monitoring in the future if the following scenarios occur:
2. If my condition improves

⎕ Yes ⎕ Sometimes ⎕ No ⎕ Unsure

1. If my condition worsens

⎕ Yes ⎕ Sometimes ⎕ No ⎕ Unsure

1. If it becomes more inconvenient to attend a polyclinic appointment

⎕ Yes ⎕ Sometimes ⎕ No ⎕ Unsure

1. If my caregiver is no longer able to manage my condition with me

⎕ Yes ⎕ Sometimes ⎕ No ⎕ Unsure

1. If I get better at using technology

⎕ Yes ⎕ Sometimes ⎕ No ⎕ Unsure

1. If technology improves to simulate a face-to-face consult better

⎕ Yes ⎕ Sometimes ⎕ No ⎕ Unsure

1. If technology improves to further protect my confidentiality

⎕ Yes ⎕ Sometimes ⎕ No ⎕ Unsure

1. If the cost of polyclinic appointments increase

⎕ Yes ⎕ Sometimes ⎕ No ⎕ Unsure

1. If the cost of tele-monitoring decreases

⎕ Yes ⎕ Sometimes ⎕ No ⎕ Unsure

1. What would make you more willing to use tele-monitoring services? Tick all that apply.

⎕ Travelling distances and cost were offset

⎕ The waiting time devoted to each appointment was reduced

⎕ It meant less time taken off work

⎕ The time between each appointment was reduced

⎕ You had to rely less on others for transport

⎕ The cost of the appointment (excluding travel costs) were reduced

⎕ My health will be improved

⎕ Not willing

Others: ____________

1. What would make you less willing to use tele-monitoring services? Tick all that apply.

⎕ You are more comfortable with a face-to-face visit with the doctor

⎕ You are concerned it will not be as good as a face-to-face visit

⎕ You have sufficient time for a face-to-face consultation with the doctor

⎕ The travel to the clinic allows you to do other things as well

⎕ You are not sure about the confidentiality of telemedicine

⎕ No concerns

Others: ___________

1. Tele-monitoring can violate my privacy

⎕Strongly disagree ⎕Disagree ⎕Neutral ⎕ Agree ⎕Strongly Agree

1. The use of communication devices seems challenging to me

⎕Strongly disagree ⎕Disagree ⎕Neutral ⎕ Agree ⎕Strongly Agree

1. Please rank your top 3 most acceptable modes of tele-monitoring, in order of preference with 1 being the most acceptable. (You may choose less than 3 options)

__ Voice Call

__ Video Call
__ Text Messaging (SMS)
__ Smartphone Application (App )
__ Email
__ Customized Smart Device (eg. Wireless Blood Sugar/Blood Pressure Monitoring Device etc.)

__ Web Portal (Website)

_ None

1. Tele-monitoring would be satisfactory compared to seeing the doctor in person.

⎕Strongly disagree ⎕Disagree ⎕Neutral ⎕ Agree ⎕Strongly Agree

1. Tele-monitoring would save money overall.

⎕Strongly disagree ⎕Disagree ⎕Neutral ⎕ Agree ⎕Strongly Agree

1. In place of what you currently pay for follow-up consultations, what is the maximum amount of money that you would be willing to pay in order to use tele-monitoring services?

⎕$0 ⎕$5 ⎕$10 ⎕$12.50 ⎕$15 ⎕$17.50 ⎕$20 ⎕$22.50 ⎕$25 ⎕$27.50 ⎕$30 Others:____________ ⎕NA

1. How much money are you willing to pay per usage of a tele-monitoring service?

____per usage ⎕ I am not willing to use tele-monitoring

1. How much of your overall healthcare cost per year do you think tele-monitoring can save?

____per year ⎕ I am not willing to use tele-monitoring

1. I will only accept tele-monitoring after seeing reports of patients benefitting from it.

⎕Strongly disagree ⎕Disagree ⎕Neutral ⎕ Agree ⎕Strongly Agree

1. I would not be able to explain my problems adequately via tele-monitoring.

⎕Strongly disagree ⎕Disagree ⎕Neutral ⎕ Agree ⎕Strongly Agree
